# Preconceptual paternal ethanol drinking induces sexually dimorphic behavioural changes across 2 generations

**DOI:** 10.1007/s00213-025-06807-w

**Published:** 2025-05-20

**Authors:** Sahir Hussain, Darren Day, Bart A. Ellenbroek

**Affiliations:** 1 School of Psychological Sciences, Te Herenga Waka - Victoria University of Wellington, Wellington, New Zealand; 2 School of Biological Sciences, Te Herenga Waka - Victoria University of Wellington, Wellington, New Zealand

**Keywords:** Alcohol, Preconceptual paternal ethanol exposure, Behaviour, Transgenerational, Sex differences, Voluntary alcohol consumption

## Abstract

This study aimed to assess both the inter and transgenerational impacts of preconceptual paternal ethanol Exposure (PPEE) using a rat model. Sprague Dawley male rats (F0) underwent chronic voluntary ethanol intake and at the end of the drinking paradigm were kept for one spermatogenesis cycle before being mated with ethanol naïve females. The litters and matched controls were behaviourally assessed, and a cohort of F1 males mated to observe a F2 generation. PPEE caused behavioural changes in both the F1 and F2 generations, including altering litter sizes and delaying development. The F1 also show a reduction in sensitivity to the motor impairing effects of ethanol compared to controls. Sexually dimorphic effects were seen with female offspring having a reduced preference to ethanol in both the F1 and F2, while tolerance to ethanol induced motor coordination was seen in the F2 females but not F2 males. Likewise, F1 males presented reductions in locomotor activity but these effects did not persist in the F2. The findings show PPEE induces transgenerational changes in development, drinking behaviour and ethanol sensitivity in a sexually dimorphic manner. These changes may be protective to the female offspring of PPEE to modify their ethanol intake. The alterations demonstrate potential far-reaching consequences for the metabolism of xenotoxic substances extending beyond ethanol and provides evidence to support developmental and behavioural changes across generations due to paternal alcohol consumption.

**Supplementary Information:**

The online version contains supplementary material available at 10.1007/s00213-025-06807-w.

## Introduction

Alcoholism and alcohol abuse remains a major health problem in society and much research focusses on uncovering the risk factors as well as the neurobiological alterations induced by (chronic) alcohol exposure. These direct impacts of alcohol also include the involuntary exposure of foetuses to alcohol during pregnancy which can lead to foetal alcohol spectrum disorders. Although much less investigated, there is mounting evidence from animal research that paternal preconceptual ethanol exposure (PPEE) can also affect the development and behaviour of the offspring. PPEE has been shown to alter gestation lengths and placental weights in dams, reductions in litter sizes, pup weights, pup viability and changes in pup sex ratios (Abel 1989, 1995; Chang et al. 2019; Klassen and Persaud 1976; Rathod et al. 2020; Stockard and Papanicolaou 1916a), although other studies found no differences (Asimes 2018; Bielawski and Abel 1997; Bielawski et al. 2002; Ceccanti et al. 2016; Chang et al. 2017; Ledig et al. 1998). In addition, alterations in locomotor activity and motor coordination in the F1 offspring, as well as in sensitivity to alcohol later in life have been reported, with several papers showing an increase in locomotor activity in the offspring of alcohol exposed sires (Abel 1993; Ledig et al. 1998). Again, a few studies found no differences in locomotor activity (Beeler et al. 2019; Nieto et al. 2022; Rompala et al. 2017). Two studies highlighted reduced drinking in ethanol sired male mice only (Finegersh and Homanics 2014; Rompala et al. 2016), and others, including our lab, showed the opposite trend with reductions seen in female offspring (Hussain et al. 2022; Rathod et al. 2020) indicating the presence of important sex differences in response to paternal ethanol drinking.

Since there are many inconsistencies in the literature on the effects of PPEE, we carried out this study using an adaptation of the intermittent access two bottle choice (IA2BC) paradigm to investigate the effects of voluntary alcohol consumption in offspring of male rats (Hussain et al. 2022; Simms et al. 2008; Spoelder et al. 2017). This model better mimics the human situation by getting rodents to develop high levels of drinking in a voluntary fashion with intermittent access as opposed to forced drinking procedures which are common practice this line of research (Asimes et al. 2018; Ceccanti et al. 2016; Finegersh and Homanics 2014; Nieto et al. 2022; Rompala et al. 2017). The reduced stress from forced practices and the voluntary nature enables us to better extrapolate our findings to help formulate public health policies to mitigate alcohol effects of PPEE. In the present study, we assess the transgenerational impacts of paternal preconceptual ethanol drinking on neurodevelopment and behaviour. To the best of our knowledge, this has not been investigated in rats, although two recent studies looked at the transgenerational impact of foetal alcohol syndrome disorders (FASD) and suggest that some behavioural changes may pass through the male germline and extend until at least the third generation (Bottom et al. 2022; Gangisetty et al. 2022). It is important to distinguish that what we propose relates to the ethanol exposure of fathers prior to conception while the transgenerational studies mentioned have looked to understand the long-lasting impacts of FASD.

It can be argued that the effects of PPEE on the first generation are not transgenerational, as the sperm used to conceive the F1 has been exposed to ethanol. We, therefore, mated the ethanol and experimentally naïve offspring of the first generation to investigate potential behavioural alterations in the second generation (F2). We hypothesised PPEE would lead to behavioural changes across multiple generations which would be in line with reports of the intergenerational impacts of paternal ethanol consumption and the transgenerational changes observed in FASD studies. Given that some of the effects of PPEE appear to be sex-dependent we included both males and female rats of the F1 and F2 generations in the study.

## Methods

### Subjects

**Fig. 1:**
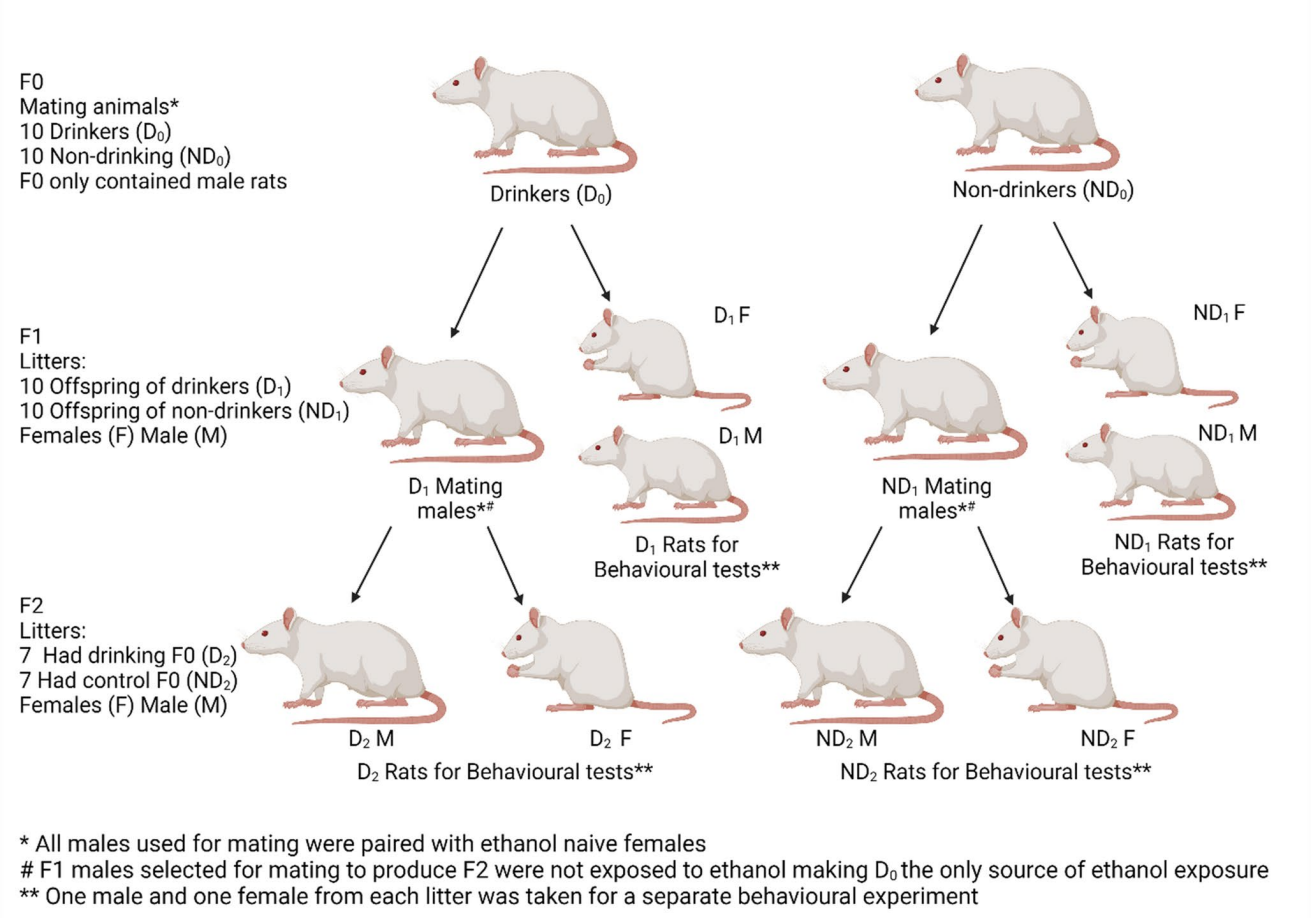
Schematic of animals in the study. Three generations of the male germline were assessed for PPEE. F1 rats used for mating were not exposed to ethanol making the D_0_ drinkers the only mated animals exposed to ethanol. Created with BioRender.com

In this study involving three generations (F0, F1, and F2) of Sprague-Dawley rats, the F0 generation comprised of 30 male and 20 control female rats. Twenty male rats were subjected to ethanol drinking through an intermittent access 2 bottle choice paradigm (IA2BC), while 10 served as matched controls with ad libitum tap water for 8 weeks. Following another eight-week alcohol-free period (to include a full spermatogenesis cycle), the 10 highest drinking males (D_0_) and the non-drinking controls (ND_0_) were mated with the 20 ethanol-naïve females (Fig. 1). Mating was done by pairing males with alcohol naïve breeding females for 21 days after which the dams were separated.

This resulted in 20 F0 pairs giving birth to 20 F1 litters, consisting of 10 offspring of drinking fathers (D_1_) and 10 offspring of non-drinking fathers (ND_1_). Litter sizes were adjusted to a maximum of 10 animals per litter on Post natal day (PND) 4, and male and female rats from each litter were chosen for behavioural experiments. Ethanol-naive F1 males were then paired with ethanol-naïve females to produce the F2 generation, assessing the potential multigenerational effects of male drinking. Seven litters with paternal grandfathers with a history of drinking (D_2_) and 7 control litters with no alcohol history (ND_2_) were obtained (Fig. 1).

All subjects were bred and reared at Victoria University of Wellington, New Zealand, in their vivarium and were kept on a reversed 12 h light/dark cycle (lights on at 7 p.m.), in a temperature (20 ± 2^o^ C) and humidity (60 ± 5%) controlled housing room. Rats were provided food and water ad-libitum for the duration of the study. Animal care procedures and experimental protocols were approved by the Victoria University animal ethics committee.

Developmental milestones were assessed at the litter level while behavioural tests were administered on different cohorts consisting of one male and one female rat from each litter to prevent influence of other test conditions. The tests included surface righting reflex at PND 2 and PND 4, locomotor activity (PND 21–23), motor coordination assessments (starting PND 35) and ethanol intake via IA2BC (Starting PND 30). A total of 36 F1 rats (*n* = 9 rats per group) and 24 F2 rats (*n* = 6 rats per group) were used in the locomotor activity and rotarod experiments. While 28 F1 (*n* = 7 rats per group) and 23 F2 (8 ND_2_M and 5 each in ND_2_F, D_2_F and D_2_M) rats underwent the IA2BC.

**Table 1:** Summary of key findings from the study

Experiment	Generation	Effects			Description
		PPEE	Sex	PPEE*Sex interaction	
Developmental milestones	F1	Yes	N/A	N/A	Sig. delay in pinnae detachment, mean delays in other milestones, increased litter variance and pup deaths in D_1_
	F2	Yes	N/A	N/A	Sig. delay in coat growth, eye opening and pup deaths, mean delays in other milestones and increased litter variance in D_2_
Surface righting	F1	No	No	Yes	At PND2, D_1_ males achieved surface righting faster than other groups.
	F2	No	No	No	No differences
Locomotor Activity	F1	No*	Yes	No	The D_1_M reared less than the other conditions, Females show greater locomotor activity than males.
	F2	No	No	No	No differences
Accelerating Rotarod	F1	Yes	No*	Yes	ND_1_ rats perform better than D_1_ under saline but D_1_ perform better when injected 1 g/kg ethanol. Females perform better than males on average.
	F2	Yes	Yes	Yes	The differences are seen due to D_2_F performing better than the other groups
Ethanol Consumption	F1	No*	Yes	Yes	The D_1_F showed reduced drinking compared to the ND_1_F. Females drank more than males
	F2	Yes	Yes	Yes	The D_2_F showed reduced drinking compared to the ND_2_F. Females drank more than males

* next to a “No” denotes trends which did not reach significance and N/A denote not applicable. Abbreviations: F0/sire condition: D (drinker), ND (non-drinker), F (female), M (male), subscripts denote generation of the rats

#### Blinding

Due to the nature of the experiment, blinding was performed where possible. Experimenters were aware of the drinking and non-drinking rats in F0, however, after the F0 set, the mating pairs and subsequent litters were coded by lab technicians to allow for the experimenter to be blinded during observation of mating and developmental tasks. After weaning, each animal was given a code tied to their litter ensuring the experimenter remained blind to the sire’s condition while running the behavioural experiments one male and female from each litter with no crossover.

### Procedures

#### Intermittent alcohol two bottle choice (IA2BC) paradigm

**Fig. 2:**
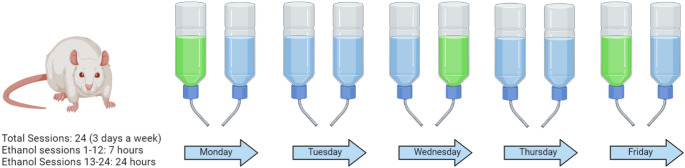
Schematic of the IA2BC drinking paradigm. A 20% (v/v) Ethanol solution was provided along with a bottle of water three times a week. Ethanol was provided for 7-hours for the first 12 sessions after which the rats were given ethanol access for 24-hours during remaining 12 session The ethanol bottle location was randomized to prevent side preference. Created with BioRender.com

All three generations of rats underwent an adapted version of the intermittent access two bottle choice paradigm (Fig. 2) as previously described in Hussain et al. (2022). Hazardous alcohol drinking behaviours are particularly prevalent during adolescence in humans along with it being a time of increased vulnerability to developing AUD (Bolland et al. 2016; Dawson et al. 2008). Similar results have been seen in rodents (Spear 2016; Varlinskaya and Spear 2004) which is why IA2BC was started when rats were aged PND 30–35.

#### Development milestones

Delays in developmental milestone achievement consistently act as predictors for larger behavioral abnormalities later in life (Ellenbroek et al. 2005; Foley et al. 2014; Smirnov and Sitnikova 2019). The few studies looking at paternal ethanol exposure on developmental milestones show mixed results (Jamerson et al. 2004; Meek et al. 2007). However, PPEE has shown effects on litter sizes, pup sex ratios, birth weights and litter viabilities (Abel 1989, 1995; Chang et al. 2019; Emanuele et al. 2001; Klassen and Persaud 1976; Ledig et al. 1998; Rathod et al. 2020). Additionally, no studies we know of have looked at early developmental milestone differences one generation removed from paternal ethanol exposure. The milestones were based on criteria previously described (Barkur and Bairy 2015; Geisler et al. 1993; Smirnov and Sitnikova 2019), and were assessed in the home cages by visual observation to avoid any undue stress to the dam or her pups. Litters were checked from PND1 to weaning at PND21 to check for the attainment of eye opening, pinnae detachment, coat growth, walking, rearing and self-grooming (see table S1 for details). The cages were viewed twice a day for 10 min each, and a milestone was said to be achieved for the litter when at least three pups being observed in the litter (30%) met the required criteria for a milestone were visible at the same time. This timing and selection criteria were chosen because of the difficulty in identifying individual pups without removing them from the cage and interrupting their activities at the time of observation. Those observing were blinded to the parental conditions of each of the litters.

#### Surface righting reflex

In addition to the observed developmental milestones, one male and one female pup at PND4 from each litter were taken for surface righting, after which they were culled for tissue collection for other studies. The pups taken from litters with over 12 pups at PND2 were also checked for surface righting before being culled.

**Fig. 3:**
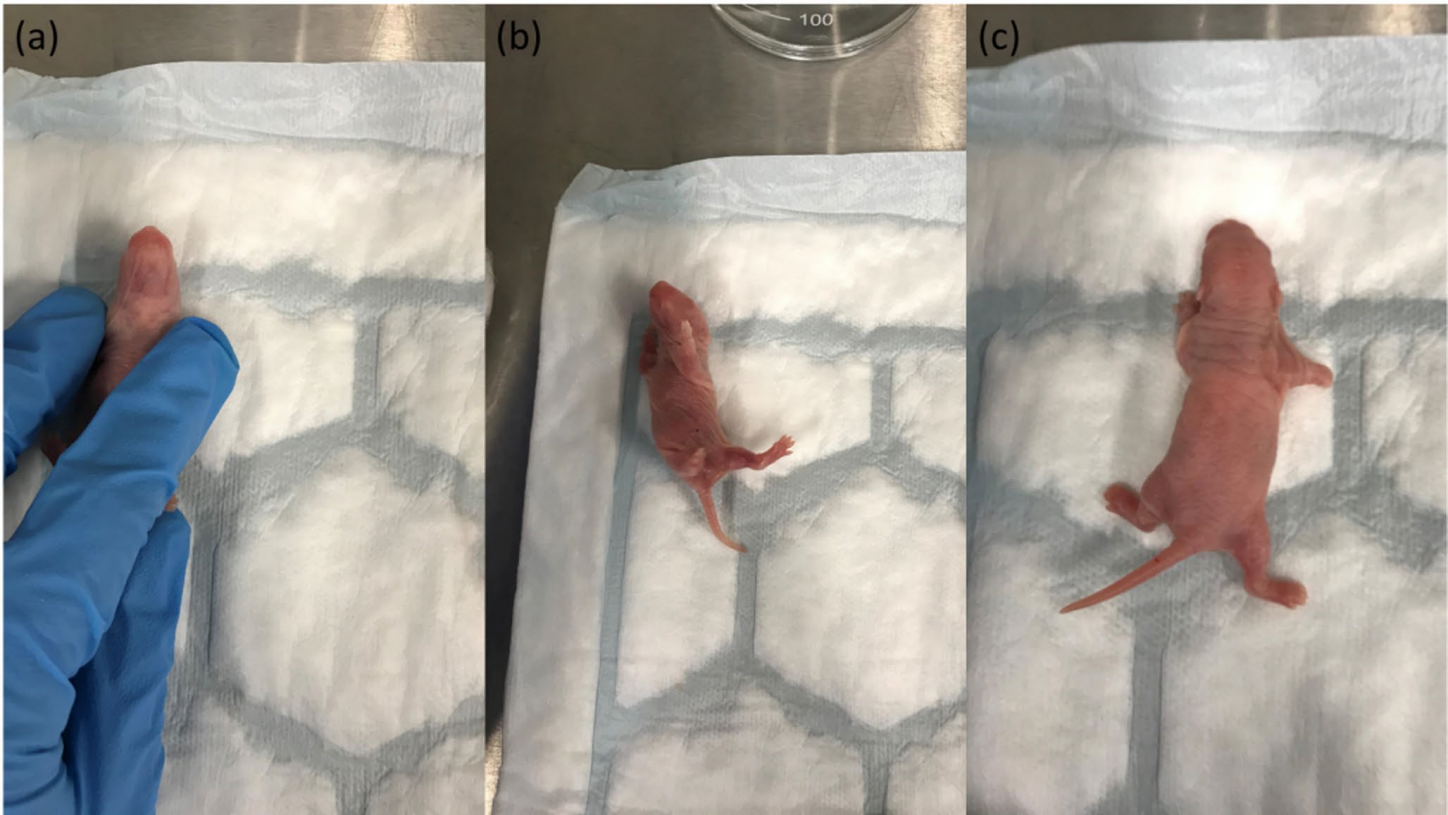
Surfacing righting procedure (**a**) Placing the pup in the supine position, timer starts as soon as experimenter takes their hand off the pup. (**b**) Rat pup tries to right itself. (**c**) Timer stops as soon as pup rights itself in the prone position

Each pup was placed in a supine position (in abnormal position, back resting on the ground surface) and was left to change itself into a prone position (all the 4 feet in contact with surface). Each pup was given three trials two minutes apart. Each pup got a maximum of 1 min per trial to be able to right itself. The shortest time to right was taken for analysis with that score acting as a representation for the remaining pups of that litter (Fig. 3).

#### Locomotor activity

Locomotor activity is often used to analyze drugs, genes, and disease models. Measuring activity in an open chamber allows for a simple assessment of general activity levels, like, distance moved, vertical counts (rearing) and times spent in pre-defined areas. The advantage of the test is that it creates discretely measurable variables that can be used to quantify the effects of different conditions. For example, acute ethanol injections show dose dependent decreases in locomotor activity (Liljequist and Karcz-Kubicha 1993; Overstreet et al. 2004; Randall, C. L. et al. 1975) and some aspects of locomotor activity are even increased under ethanol exposure (Correa et al. 2003; Frye and Breese 1981).

The chamber was a square arena mounted with two planes of infrared detectors in a specially designed sound attenuating Plexiglas chamber (Med Associates, W×43.2 × 43.2, H×30 cm). Rats were introduced to the testing room 10–15 min before to get habituated to the new surroundings. An animal was then placed into the centre of each chamber and activity was recorded for 60 min and processed by a computer program (Activity Monitor Version 5 program: Med Associates Inc., St Albans, VT USA). Horizontal locomotor activity and vertical movements (rearing) were detected by rats breaking the infrared beams across the arena. The testing room was dimly lit by a red light and a white-noise generator was used to reduce extraneous noise disturbance. On completion, rats were placed back in their home cages and returned to the home room. The apparatus was then cleaned and dried in preparation for the next rat. Testing was done on 3 consecutive days when the rats were aged PND22-24.

#### Accelerating Rotarod

The test is based on a rotating rod with forced motor activity being applied, by a rodent. The test measures parameters such as riding time (seconds) or endurance. The main functions of this test are to evaluate balance, grip strength and motor coordination of the subjects; especially in testing the effect of experimental drugs (Rustay et al. 2003; Uzbay and Wallis 1999). The length of time that a given animal stays on this rotating rod is a measure of their balance, coordination, and physical condition. The experiments were performed using an accelerating Rotarod treadmill (Rotarod LE 8500 (76–0239), L x 50 cm, H x 36 cm, W x 24 cm, Panlab, Barcelona, Spain). With procedures used a being the same as described in Hussain et al. (2022).

### Data analysis

Statistical analysis was carried out using GraphPad Prism (version 8.0.2 for Windows). Independent t-tests and ANOVAs (Analysis of Variance) were used to compare the developmental milestones and surface righting reflex across the conditions. A generalized linear mixed model analysis with an autoregressive covariance structure was used for ethanol consumption. This model was chosen to accommodate for days during which data of a subject was lost due to leaks in the drinking bottles (Wang and Goonewardene 2004). Ethanol preferences were averaged across the 7-hour sessions, 24-hour sessions and total drinking days for different analyses of preference. The ethanol-induced motor-coordination across 3 injections were measured using mixed factorial ANOVAs, as no data points were missing. There was no difference in performance between the 10-min post-injection and the 30-min post injection trials and so the best time for each rat was taken. Mixed factorial ANOVAs were also used for locomotor activity. Levene’s test was used to verify the assumption of homogeneity of variance across groups and Tukey’s Honest Significant Difference (HSD) test was used as post hoc analysis for the ANOVAs which showed significant differences.

## Results

### Drinking acquisition in F0

The mean g/kg ethanol consumed across all the drinking sessions was 3.60 g/kg (*SD* = 1.10 g/kg). Repeated measures ANOVAs showed the success of the drinking paradigm with a significant increase in drinking across days (*F* (23, 391) = 16.9, *p* < .001). Alcohol preference across all the days averaged 29% (*SD* = 8.18%) and varied with the rat with the highest preference to ethanol being showing a 47.4% preference and the lowest preference being 16.9% (Fig. 12). All 20 rats in the study developed substantial alcohol drinking, confirming the effectiveness of the intermittent access paradigms for the acquisition of consistent drinking behaviours (Figure S1).

### Developmental milestones

As developmental milestones were observed in the home cage, comparisons could only be made on a litter-by-litter basis in the F1 and F2. Litter size at PND0, coat growth, eye opening, pinnae detachment, walking, self-grooming and unsupported rearing were compared (Table S1).

**Fig. 4:**
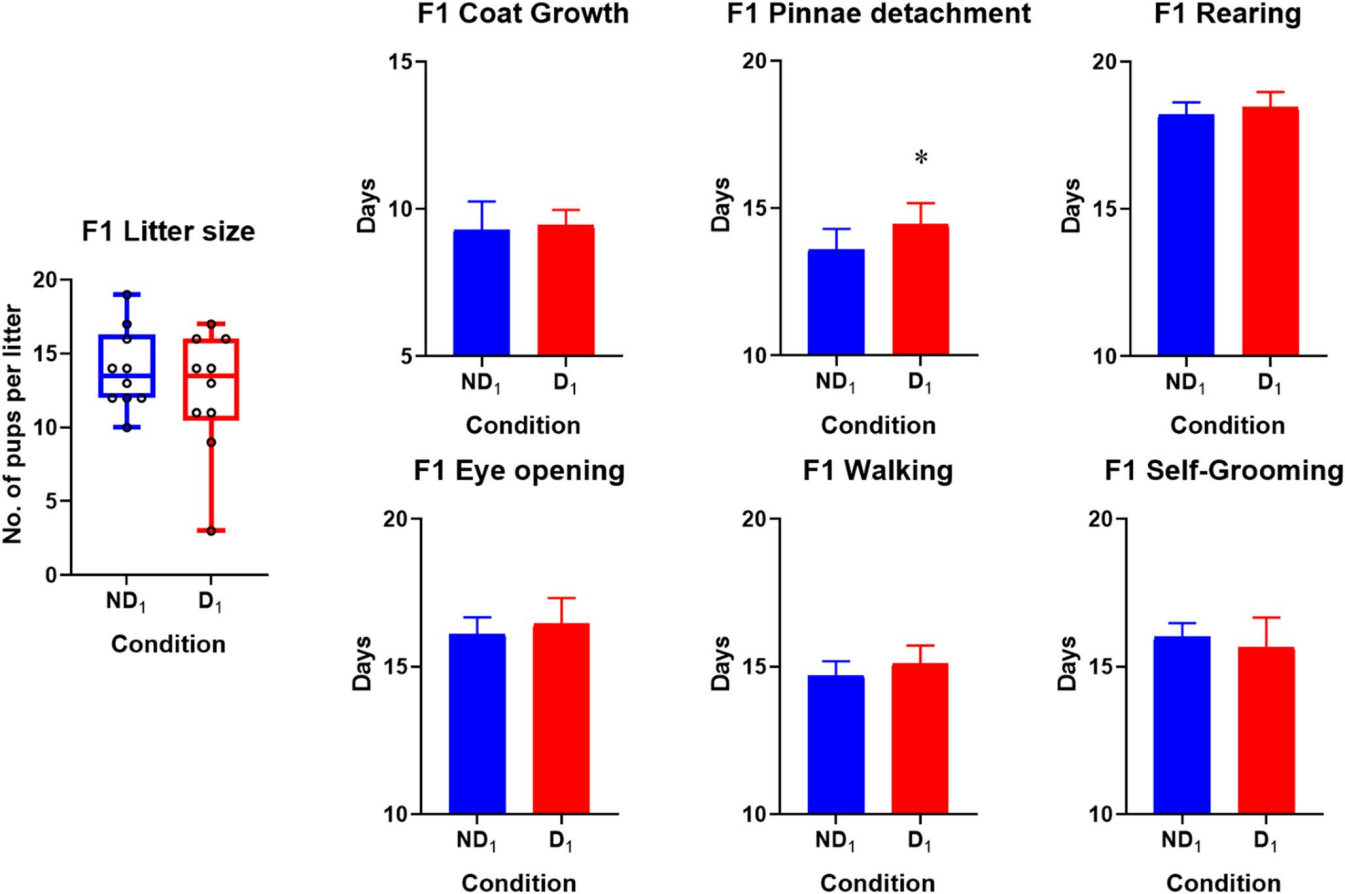
Litter sizes at birth and developmental milestones in F1 litters. The day at which milestone achieved on the Y-axes. Pinnae detachment showed a significant delay in offspring of drinkers compared to non-drinking sires (*t* (17) = 2.58, *p* = .019). Litter sizes showed high variance in D_1_ and eye opening, walking and rearing were also slightly delayed in the D_1_. Bars denote standard deviations, Abbreviations: D (drinker), ND (non-drinker), F (female), M (male), subscripts denote generation. * Denotes significant difference

**Fig. 5:**
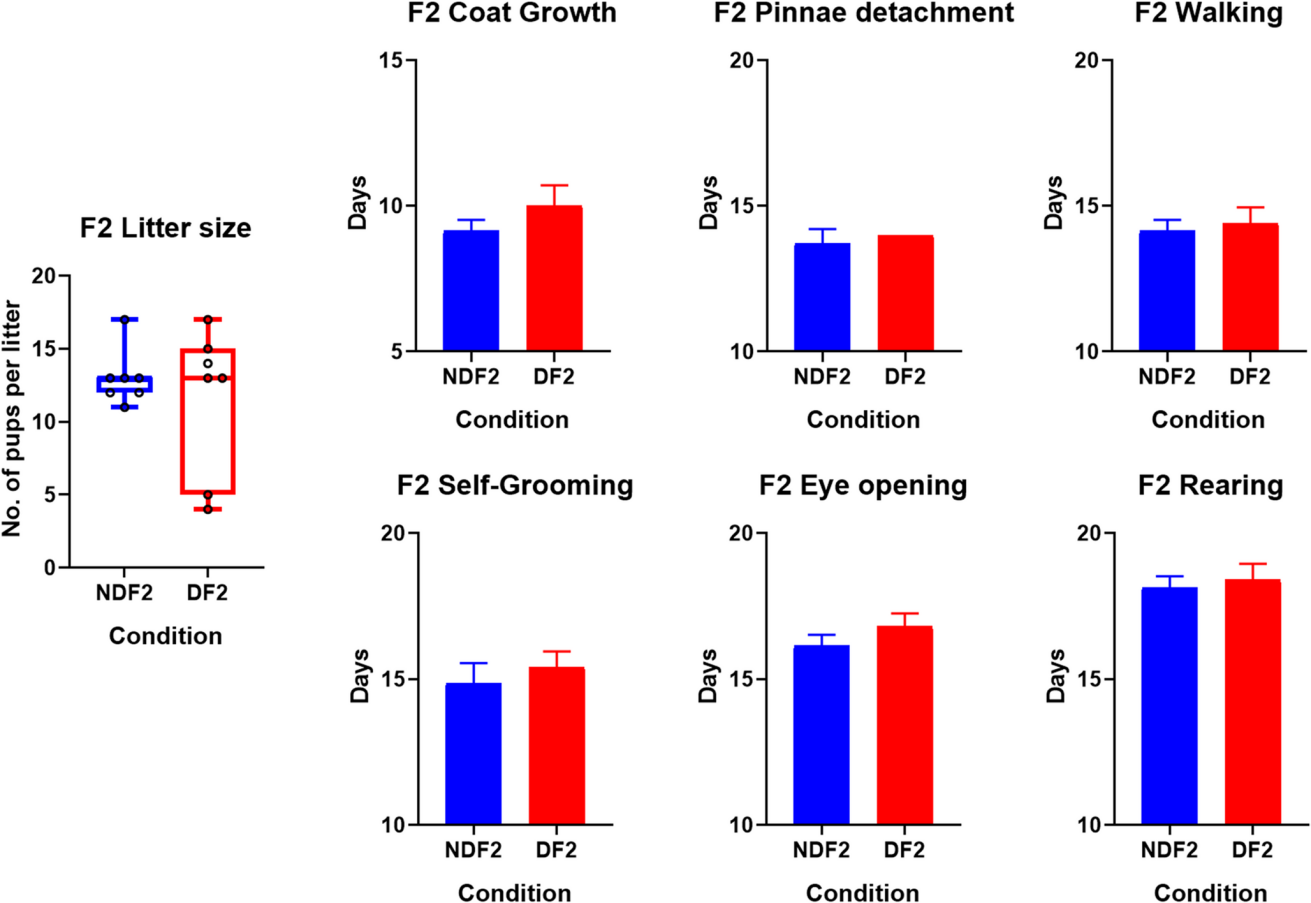
Litter sizes at birth and developmental milestones in F2 litters. The day at which milestone achieved on the Y-axes. Coat growth (*t* (10) = 2.74, *p* = .021) and eye opening (*t* (10) = 2.76, *p* = .020) showed a significant delay in offspring of drinkers compared to non-drinking sires. Again, the variance in litter size was high in the D_2_ and all other milestones showed slight delays. Abbreviations: D (drinker), ND (non-drinker), F (female), M (male), subscripts denote generation. Bars denote standard deviations, * Denotes significant difference

The mean litter sizes were larger in the ND compared to the D in both the F1 and F2 but this was not significant. However, D1 and D2 both had notably larger variances when compared to ND litters (Figs. 4 and 5). One D_1_ litter and two D_2_ litters did not survive till the measurements of developmental milestones. D_1_ litters also had 8 deaths, split across 2 litters, between PND0 and PND4 unrelated to the culling while ND_1_ litters only had 2 such occurrences. The D_2_ litters had 6 deaths between PND0 and PND4 while none were recorded in the ND_2_. Another thing to note is that, while we did not assess gestation days, the study had the mating pairs together for a fixed duration of time of 21 days giving a limited window for conception to occur. The naïve females mated with drinkers (D_0_) and Drinker offspring (D_1_) gave birth later than those mated with the ND_0_ and ND_1_. Suggesting there may be differences related to mating, fertilization and gestation due to sire ethanol consumption (Table S1). However, the current study did not assess specific factors in the mated males or females relating to analysis of vaginal plugs, smears or sperm quality and therefore cannot make any further inferences.

Although most developmental milestones were delayed in the D litters, only the differences in pinnae detachment (*t* (17) = 2.58, *p* = .019) reached significance in the F1, while coat growth (*t* (10) = 2.74, *p* = .021) and eye opening (*t* (10) = 2.76, *p* = .020) were significantly delayed in D_2_ litters compared to ND_2_ (Figs. 4 and 5). When comparing doing cross-generational comparisons, there was no significant difference of litter size, coat growth, rearing, eye opening and pinnae detachment. However, walking, and self-grooming had significant generation effects where F2 showed walking (*F* (1, 27) = 11.13, *p* = .002) and self-grooming (*F* (1, 27) = 6.89, *p* = .014) sooner than the F1 rats. Lastly, Eye opening (*F* (1, 27) = 4.54, *p* = .042) and pinnae detachment (*F* (1, 27) = 6.19, *p* = .019) showed a significant effect of sire condition only. These suggest that while some developmental delays remain others might improve a generation removed from PPEE.

### Surface righting reflex

**Fig. 6:**
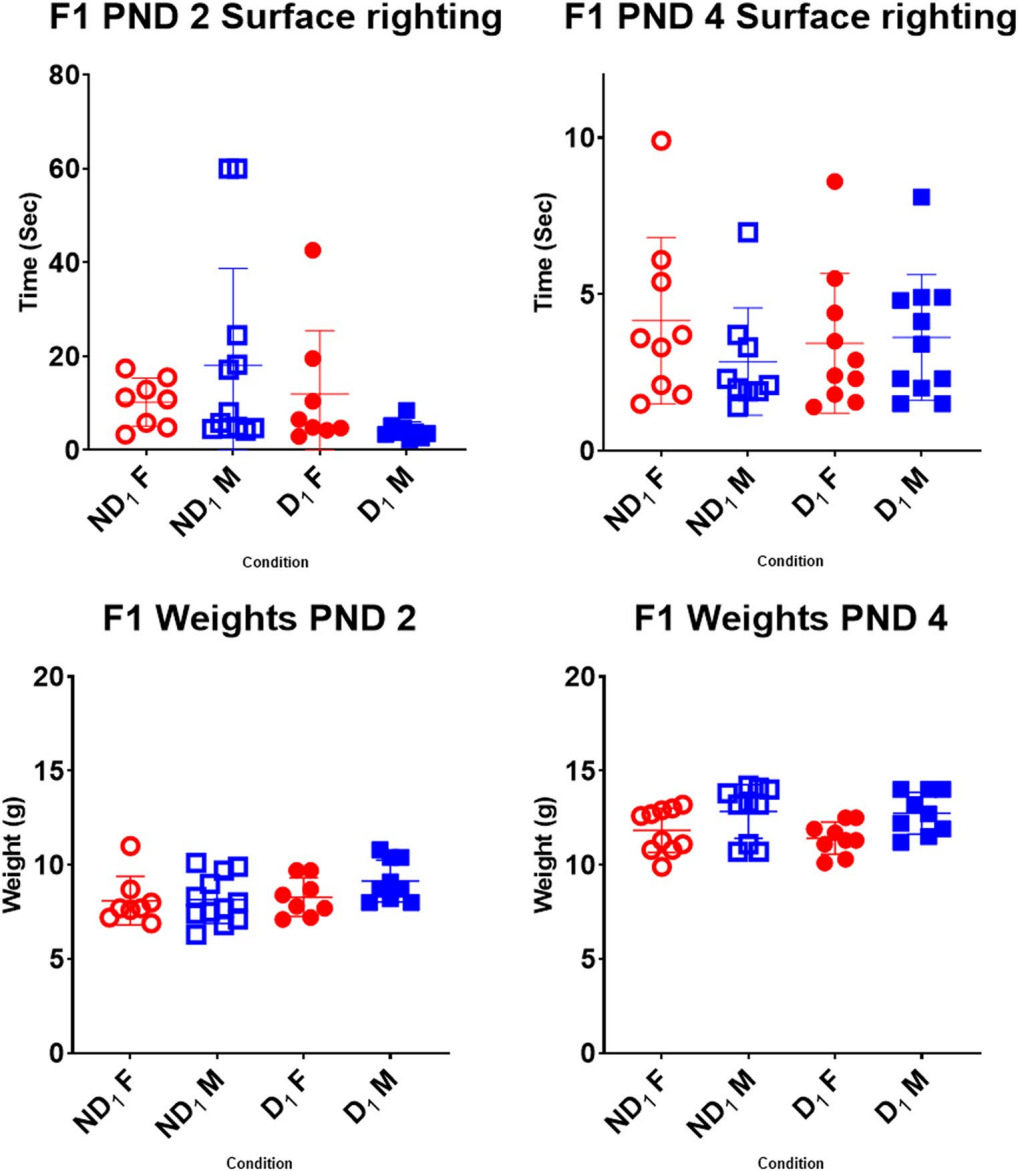
Comparison of surface righting times and weights in the F1. Age (*F* (1, 67) = 190.93, *p* < .001) and sex (*F* (1, 67) = 8.75, *p* = .004) showed significant differences with PND 4 pups weighing more than PND 2 pups and males weighing more than females. While looking at surface righting times, there was a significant three-way parent * sex * age interaction (*F* (1, 69) = 4.42, *p* = .039) where surface righting was slower in PND 2 ND_1_ males compared to the others, but the difference was not observed at PND 4. Abbreviations: D (drinker), ND (non-drinker), F (female), M (male), subscripts denote generation. Bars denote standard deviations

**Fig. 7:**
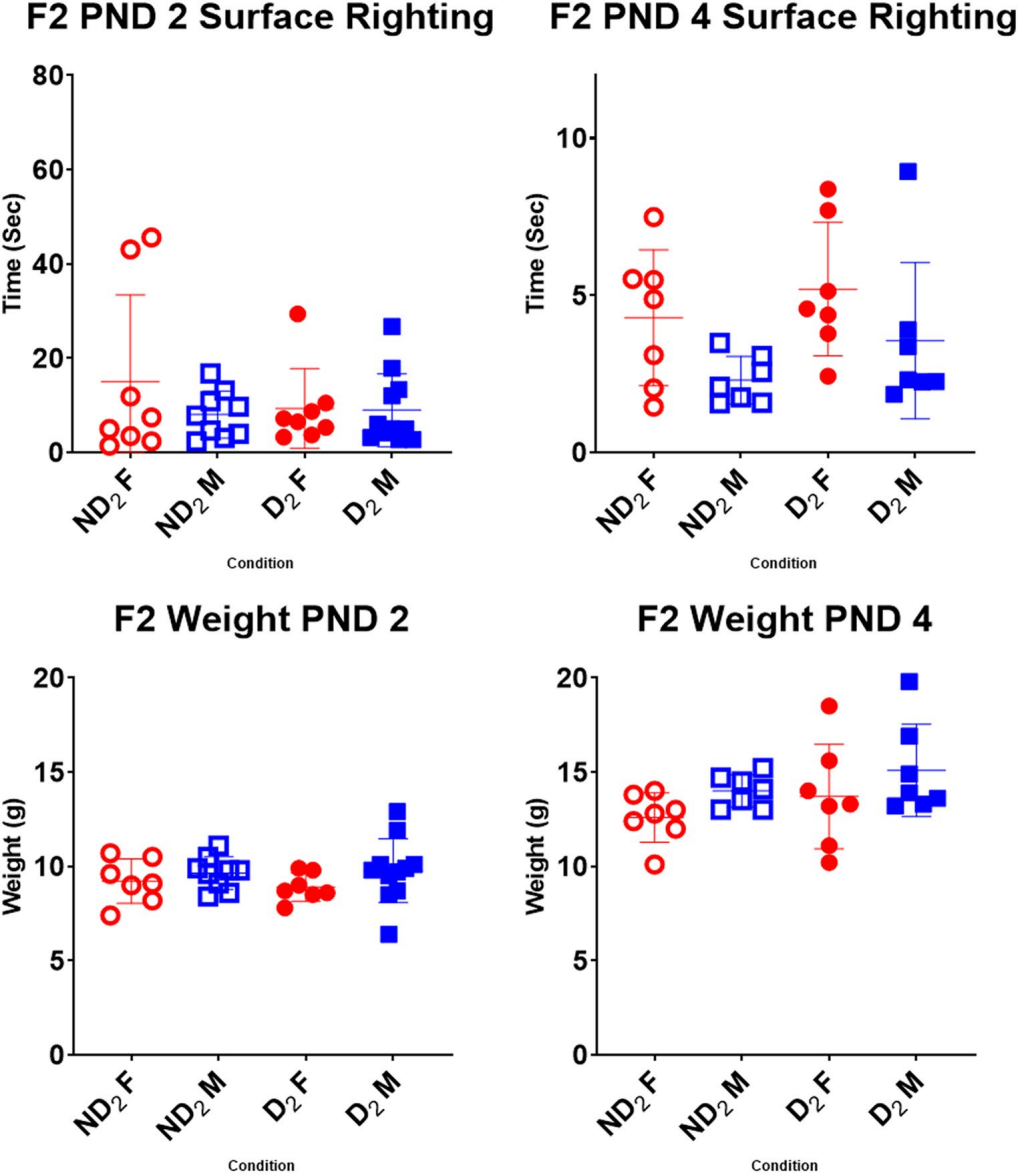
Comparison of surface righting times and weights in the F2. When looking at weights, age (*F* (1, 54) = 113.68, *p* < .001) and sex (*F* (1, 54) = 6.00, *p* = .018) showed significant differences with PND4 pups weighing more than PND2 pups and males weighing more than females. Surface righting improved with age (*F* (1, 54) = 9.87, *p* = .003) but there were no other significant effects. Abbreviations: D (drinker), ND (non-drinker), F (female), M (male), subscripts denote generation. Bars denote standard deviations

Differences for weight and surface righting ability were compared in ANOVAs by age (PND 2/PND 4) x sex (male/female) x sire condition (D/ND). In both the F1 and F2 rats, weights, age and sex showed significant differences with PND 4 pups weighing more than PND 2 pups and males weighing more than females (Figs. 6 and 7). There was no effect of parental condition on weights.

While looking at surface righting times, age again showed a significant effect with latencies reducing from PND2 to PND4 in both generations (Table S2). Lastly, in the F1s, there was a significant three-way parent * sex * age interaction (*F* (1, 69) = 4.42, *p* = .039) where surface righting was slower in PND2 ND_1_ males compared to the others, but the difference was not observed at PND4 (Fig. 6). This effect was not seen in the F2 (Fig. 7). There were no significant differences when comparing across generations.

### Locomotor activity

**Fig. 8:**
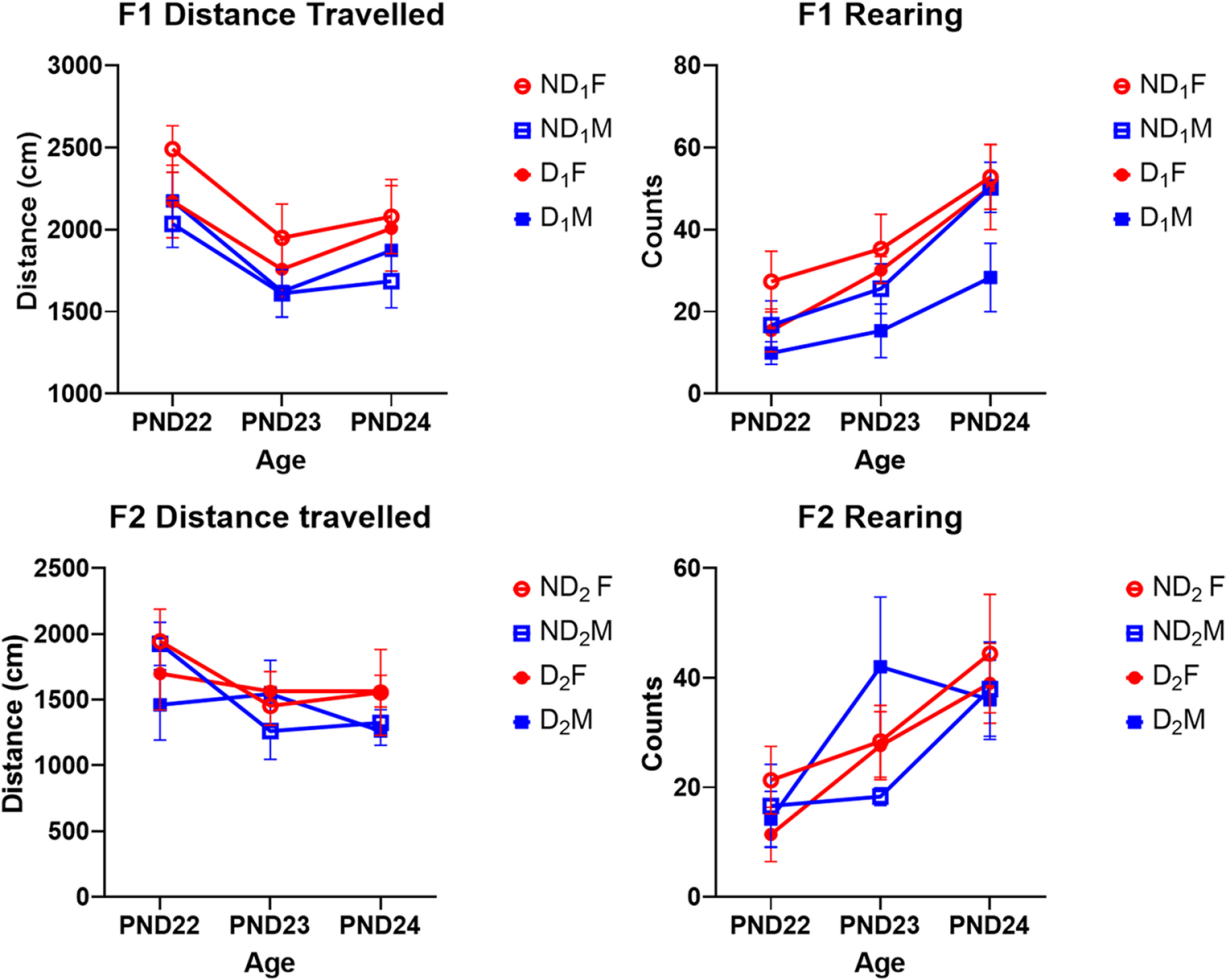
Distance travelled and rearing of the F1 and F2 rats in the locomotor activity chamber. Distance travelled reduced over the days while rearing behaviour increased. While the F1 travelled more than the F2 (*F* (1, 52) = 14.13, *p* < .001). F1 females travelled further and reared more than the males on average (*F* (1, 32) = 4.92, *p* = .034). ND_1_ showed more rears than D_1_ (*F* (1, 32) = 3.94, *p* = .056) with this being due to the D_1_ males. F2s showed no differences through either sex or parent. Abbreviations: D (drinker), ND (non-drinker), F (female), M (male), subscripts denote generation

Measurement of locomotor activity in both F1 and F2 generations showed a significant change in distance travelled, ambulatory counts and rearing across the testing days with distance travelled and ambulatory counts decreasing from PND 22 to PND 24 (Table S3). The opposite was seen in rearing counts, with an increase from PND 22 to PND 24. In the F1, there was a significant effect of sex when looking at rears, with females showing more counts of rearing than males (*F* (1, 32) = 4.92, *p* = .034). The ND_1_ group on average had more rears than the D_1_ but this did not reach statistical significance, however, the effect is almost entirely down to the low number of rears seen by the D_1_ males (Fig. 8). Lastly, females on average also travelled more distance than males but this was not statistically significant (*F* (1, 32) = 3.94, *p* = .056). There were no significant effects of either sex or F0 drinking condition in the F2s for locomotor activity indicating a potential fading of the impacts of PPEE for the phenotype. Behaviours remained similar across generations, there was a significant difference in distance travelled between generations with the F1 rats travelling more than the F2 (*F* (1, 52) = 14.13, *p* < .001).

### Accelerating Rotarod

**Fig. 9:**
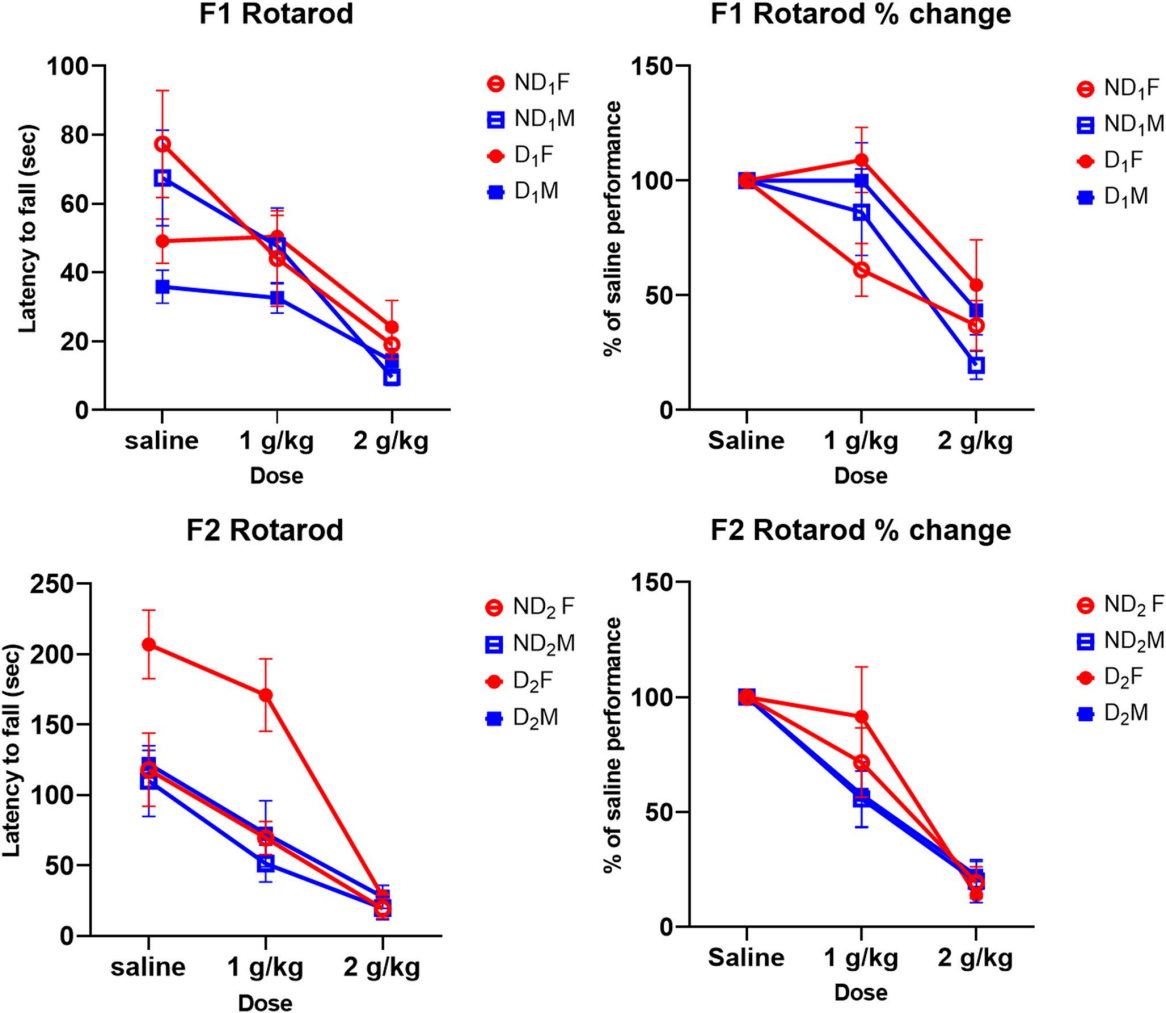
Average latency to fall (in seconds) and percentage drop in performance on the rotarod. A clear effect of dose is seen in both generations with performance dropping with increase in ethanol dose. Females perform better than males in both the F1 and F2 rats (*F* (1, 52) = 15.82, *p* < .001). ND_1_ animals perform better on the saline than the D_1_ rats but they also show a higher percentage drop in performance (*F* (1, 32) = 6.07, *p* = .019). The F2 D_2_ females perform significantly better than the other groups while the D_2_ males perform similar to the non-drinkers indicating sexually dimorphic transgenerational effects. Abbreviations: D (drinker), ND (non-drinker), F (female), M (male), subscripts denote generation

Rats were compared on their latency to fall and their percentage performance on the ethanol dose compared to saline. Here, the independent variables were sex, sire (father drinking) condition and ethanol dose. There was a significant effect of dose with a reduction in performance with increasing doses of ethanol in both F1 (*F* (2, 64) = 26.46, *p* < .001) and F2 (*F* (2, 40) = 53.70, *p* < .001). Tukey’s post hoc analysis in F1 and F2 generations showed significant differences between saline and both 1 g/kg (*p* < .008) and 2 g/kg (*p* < .001) dose while there was also a significant difference between the 1 and 2 g/kg doses (*p* < .001). Females performed better than males in both F1 and F2 (Fig. 9).

In the F1, there was a significant dose x sire condition interaction (*F* (2, 64) = 6.62, *p* = .002) with the ND_1_ animals performing better on the saline than the D_1_ rats, however this difference disappeared after ethanol administration (*p* > .900). F1 Rotarod performance as a percentage of the performance on saline showed no difference between males and females in terms of change in performance across doses but there was a significant main effect of sire drinking condition where the performance drop was blunted in the D_1_ condition (*F* (1, 32) = 6.07, *p* = .019).

**Fig. 10:**
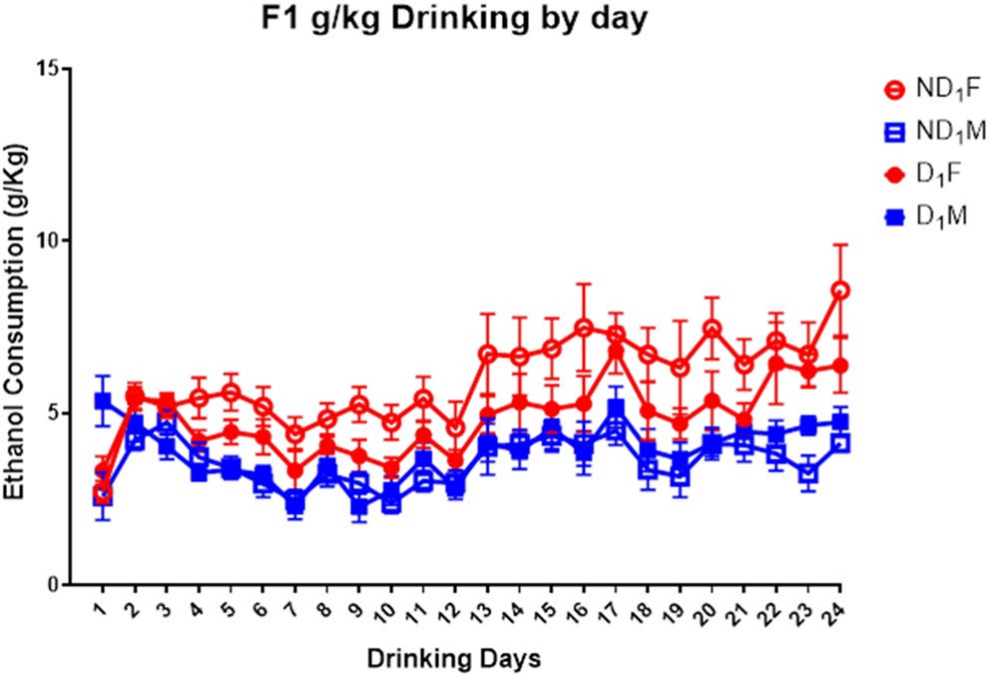
Daily g/kg ethanol intake of the F1 cohort. The first 12 sessions had a 7-hour access to ethanol while rats had 24-hour ethanol access for sessions 13–24. Females drank significantly more than males (*F* (1, 81.452) = 60.95, *p* < .001) and there was a sex x parent condition interaction where the ND_1_ females also consuming significantly more than the D_1_ females (*F* (1, 81.45) = 10.96, *p* = .001)

For the F2, there was also a main effect of sire condition where D_2_ performed better than ND_2_ (*F* (1, 20) = 12.27, *p* = .002). There was also a significant sire x sex interaction (*F* (1, 20) = 5.38, *p* = .031) indicating D_2_ females show an attenuated response to the motor effects of ethanol while this is not seen in the D_2_ males (*p* = .011), ND_2_ males (*p* = .003) and ND_2_ females (*p* < .001). Lastly there was a dose x sex interaction where females performed better than males on 1 g/kg (*p* = .016) but not on saline or the 2 g/kg dose (Fig. 10). This was moderated by the performance of the D_2_ females. However, when looking at performance as a percentage of saline, the only significant effect was that of dose (Table S4).

Across the generations, there was a sex x sire condition interaction (*F* (1, 52) = 7.46, *p* = .009) where females of the drinker sires significantly outperformed all the other conditions (*p* < .002). However, this difference is mainly carried by the performance of the D2 females. Looking at the percentage drop, offspring of drinkers performed better on ethanol than offspring of non-drinkers (*F* (1, 52) = 4.21, *p* = .045) suggesting clear impacts of PPEE on motor coordination under the influence of ethanol lasting across generations.

### IA2BC

#### Ethanol intake

First, a repeated measures ANOVA assessing total daily ethanol intake showed a significant (*F* (23, 621) = 58.90, *p* < .001) increase in total ethanol drinking across days. A 2 (sex) x 2 (sire condition) x 24 (drinking days) linear mixed model analysis looking at ethanol consumption (g/kg) showed a significant effect of days, post hoc analysis indicates that drinking to bodyweight remained mostly stable with a few significant day differences (Fig. 10). In addition, there were main effects of sex (*F* (1, 81.452) = 60.95, *p* < .001), with females consuming higher amounts of ethanol. There was a near significant main effect of sire condition (*p* = .058). Importantly, there was a significant sire x sex interaction (*F* (1, 81.45) = 10.96, *p* = .001) with post hoc analysis showing that ND_1_ females consumed significantly (*p* < .05) higher amounts of ethanol per kg body weight when compared to D_1_ females and the ND_1_ and D_1_ males (Fig. 12). The other three conditions were not significantly different from each other.

**Fig. 11:**
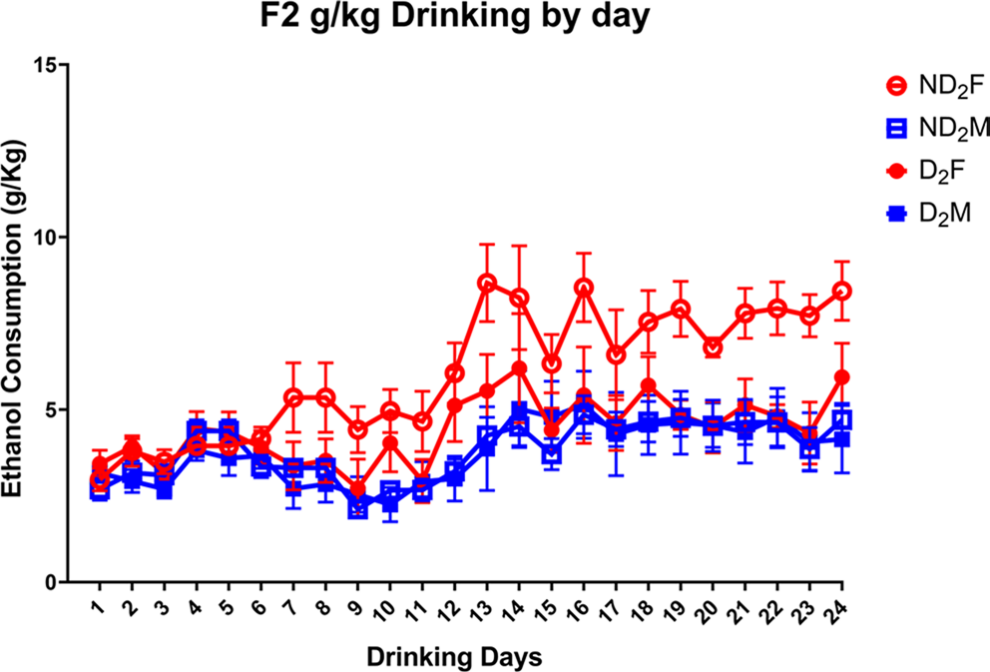
Daily g/kg ethanol intake of the F2 cohort. The first 12 sessions had a 7-hour access to ethanol while rats had 24-hour ethanol access for sessions 13–24. Females again, drank significantly more than males (*F* (1, 49.76) = 24.81, *p* < .001) with the ND2 females consuming significantly more than the other 3 groups (*F* (1, 49.76) = 7.25, *p* = .010)

Like the F1 animals, the F2 cohort showed a significant increase (*F* (23, 506) = 49.10, *p* < .001) in total ethanol intake across the drinking days. A similar linear mixed model analysis looking at ethanol consumption per kg body weight again showed a significant effect of drinking days (Fig. 11). The same main effect of sex with females consuming higher amounts of ethanol was seen F (1, 49.76) = 24.81, *p* < .001. Unlike in the F1s, there was also a main effect of sire condition with ND_2_ rats consuming more ethanol than D2, this effect was moderated by a significant condition x sex interaction (*F* (1, 49.76) = 7.25, *p* = .010), where post hoc analysis showed ND_2_ females consumed significantly higher amounts of g/kg ethanol (*p* < .05 for all) when compared to D_2_ females, D_2_ males and ND_2_ males, while there was no significant difference between the other 3 conditions (Table S5).

#### Ethanol preference

**Fig. 12:**
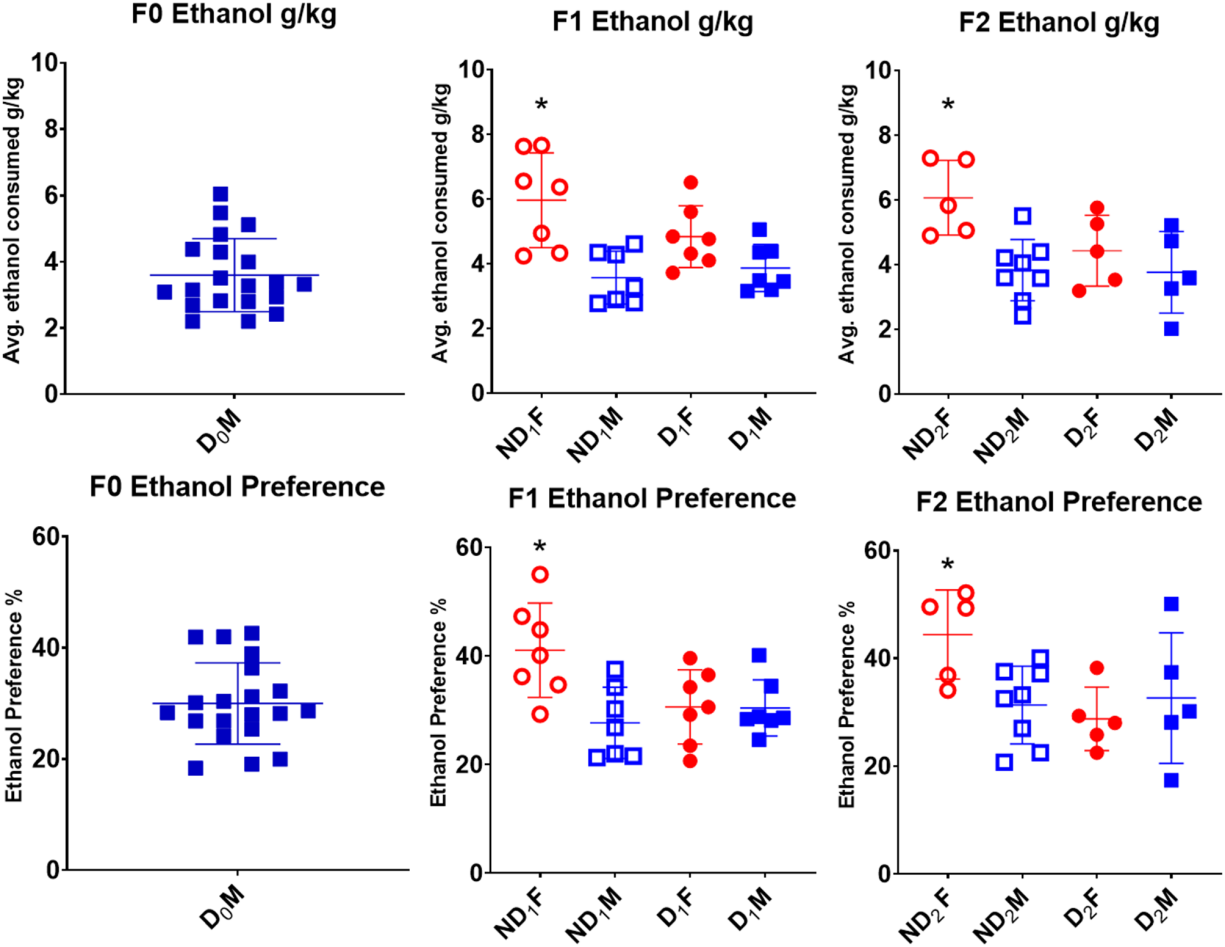
Average g/kg ethanol intake and ethanol preference of the F0, F1 and F2 cohorts. Females on average consumed more ethanol g/kg than the males and showed a greater preference to ethanol. Those sired from the ND condition showed a greater g/kg consumption of ethanol (*F* (1, 43) = 4.40, *p* = .042) and a greater ethanol preference (*F* (1, 43) = 6.41, *p* = .015) than those sired from D_0_ males. Post Hoc analysis using Tukey’s HSD showed ND females had significantly higher ethanol intake (*p* < .015) and preference (*p* < .005) compared to all other groups. Abbreviations: D (drinker), ND (non-drinker), F (female), M (male), subscripts denote generation. * Denotes a significant difference

To assess ethanol preference, a 2 (sex) x 2 (drinking condition) x 2 (drinking sessions) mixed factorial ANOVA looking at ethanol preference showed a significant effect of drinking sessions with a reduction in preference from the 7-hour drinking sessions to the 24-hour drinking sessions. There was a significant main effect of sex where F1 females showed a greater preference to ethanol than F1 males (Fig. 12). There was also a significant drinking condition x sex interaction (*F* (1, 24) = 6.36, *p* = .019) where female ND_1_ showed the highest levels of ethanol preference and was significantly different from ND_1_ males (*p* = .007), D_1_ males (*p* = .039), and D_1_ females (*p* = .043).

Unlike the F1s there was no difference in preference between the two sessions in F2 (*p* = .677). There was no significant main effect of sex (*p* = .113) or parent (*p* = .064). However, there was again a significant sire x sex interaction (*F* (1, 19) = 5.01, *p* = .037) where female ND_2_ showed the highest ethanol preference which was significantly higher than the other 3 conditions (*p* < .05) while there were no significant differences between ND_2_ males, D_2_ males, and D_2_ females (Fig. 12).

## Discussion

The results show clear developmental and behavioural differences in the offspring of drinkers which were transgenerationally inherited through the male germline to at least the second generation even if ethanol exposure is stopped 8-weeks prior to mating (Table 1). One of the most unique aspects of this study was identifying true transgenerational effects of PPEE with D_1_ and D_2_ litters having delayed development, larger litter size variances and greater premature pup death. These findings might partially be explained by studies which report changes in fertility and foetal reabsorption rates of females bred with alcohol treated males (Abel 1995; Klassen and Persaud 1976). Early developmental milestones such as eye opening, pinnae detachment and coat growth were reached later in the offspring of drinkers (D_1_ and D_2_) and compared to the non-drinker offspring (ND_1_ and ND_2_) indicating deficits in neurodevelopment, as reported by others (Meek et al. 2007). These early developmental deficits indicate neocortical development delay with abnormal gene expression patterns in the neocortex of offspring of ethanol exposed male mice (Bottom 2021; Conner et al. 2020). The study indicates some of the developmental differences reduce across generations, others remain, and new significant effects appear. The mechanisms for these are poorly understood with one suggestion being PPEE and other paternal toxic exposure causes reduced testosterone levels accompanied by epigenetic changes in placenta and sperm cells of offspring which may lead to developmental deficiencies in future generations which might not align with those seen in previous generations (Abel and Lee 1988; Abel 1989; Viluksela and Pohjanvirta 2019). One caveat to remember is that, as mentioned, the differences seen here were compared using a whole litter approach to reduce stress on the pups which reduced statistical power and could play a role in why we see significant effects in one generation and not in the other. The ability to look at sex differences is also hindered by this approach which means a pup specific approach is required to further elucidate the results observed.

Sex dependent differences were seen in the surface righting reflex with the male D_1_ pups at PND 2 having faster righting compared to the ND_1_. The finding is in line with one study which found PPEE pups show earlier attainment of surface righting compared to controls but poorer balance and motor coordination which is thought to be linked to thickening of cortical layers found in pups of ethanol exposed rats (Jamerson et al. 2004). The differences were not seen in the F2. The offspring of drinkers showed a blunted response to ethanol for motor coordination tasks compared to offspring of non-drinking sires which has previously been reported. This is supported by previous studies which have seen a reduction in ethanol sensitivity in offspring of ethanol sired animals on the rotarod (Finegersh and Homanics 2014; Hussain et al. 2022). Unlike surface righting and locomotor activity, when looking at the F2, female offspring of drinkers still showed a reduction in the impact of ethanol on motor coordination. This effect was not seen in the F2 males indicating a sexual dimorphism in transgenerational impacts on motor behaviour.

The transgenerational results were consistent with respect to ethanol drinking in the IA2BC. Female offspring in both the D_1_ and D_2_ showed reduced ethanol consumption and preference when compared to offspring of non-drinkers. Previous studies have yielded mixed results with some in mice reporting male offspring of alcohol-exposed sires show reduced alcohol intake (Finegersh and Homanics 2014; Rompala et al. 2017). Others report, only male offspring of ethanol exposed sires present decreased alcohol seeking behaviours (Campbell et al. 2018; Ceccanti et al. 2016). However, like our study, some did see ethanol-sired females having reduced drinking compared to controls (Hussain et al. 2022; Rathod et al. 2020). The differences may be attributed to a few factors such as differences in animals and strains used, different durations of paternal ethanol exposure, and ages at which the drinking and preference tests were performed.

The sex specific differences seen in surface righting, locomotor activity and ethanol drinking along with the rotarod where effects of ethanol were seen in both male and female D_1_ rats indicate certain pathways may be affected in both sexes while other changes due to PPEE might be sex specific. This idea is further strengthened by the transgenerational impacts also being sexually dimorphic and being seen in certain behaviours but not others. The sex differences, particularly of ethanol intake of female rats, could potentially be an indication of enhancement in the rewarding properties of ethanol as other studies have reported heightened response to ethanol in dopamine pathways due to ethanol in females compared to males (Blanchard et al. 1993; Blanchard and Glick 2002). In addition, serotonin reuptake transporter (SERT) knockout models show sexually dimorphic interactions with ethanol with females that have reduced SERT activity (heterozygous and complete knock out) consuming significantly more ethanol than the wild-type SERT with this effect not being seen in males (Hussain et al. 2022). Taken together, PPEE might affect these reward pathways differently in the frontal cortex leading to lower ethanol intake for achievement of the same reward in females and therefore reduced drinking. At the same time, the similar effects seen in F1 ethanol induced motor coordination in both sexes may be due to PPEE having similar impacts on the cerebellum, which is a key region for motor coordination (Manto et al. 2012).

Another reason for this dimorphism could be the difference in alcohol metabolism with rodent females having similar blood ethanol concentrations despite greater ethanol intake (Pirino et al. 2022; Randall, P. A. et al. 2017) compared to males. This may be explained by females showing a higher peak blood ethanol concentration and faster elimination rates (Crippens et al. 1999; Thomasson 1995). This is mirrored in humans with women showing greater clearance of ethanol per kg lean body mass (Kwo et al. 1998) but having significantly lower lean body mass than males in addition to significantly slower gastric alcohol metabolism with lesser alcohol dehydrogenase (ADH) activity compared to men (Baraona et al. 2001; Frezza et al. 1990) and males also show different neurobiology response to alcohol (Blanchard et al. 1993; Flores-Bonilla and Richardson 2020). Females require lower ethanol doses than males for reinforcement (Hauser et al. 2019) as the female hormone, estradiol, appears to enhance the rewarding effects of alcohol (Hilderbrand and Lasek 2018).

These differences support previous work which indicate the persistence of litter effects and early development delays across generations due to PPEE (Stockard and Papanicolaou 1916a, b). Govorko et al. (2012) and Gangisetti et al. (2022) have shown that changes due to foetal alcohol spectrum disorder (FASD) can be passed down through the male germline and human studies have seen grandmothers of children with partial foetal alcohol syndrome having histories of alcohol abuse, suggesting transgenerational effects of FASDs (Kvigne et al. 2008). While research on paternal transgenerational effects is scarce, one study on heroin exposure in Sprague Dawley sires saw both F1 and F2 male offspring had increased anxiety-like behaviours as well as increased aggression (Farah Naquiah et al. 2016). The study however did not include females which means we cannot tell whether different drugs of abuse also show sexually dimorphic transgenerational effects. Human studies have long identified trends of transgenerational epigenetic inheritance which means specific rodent studies are required to identify the underlying mechanisms especially since reports in transgenerational studies on diet have found persistence of behavioural phenotype changes despite the epigenetic markers being wiped out (Bottom et al. 2022; Gapp et al. 2014; Radford et al. 2014).

Finally, there is growing evidence to suggest there are sex-specific differences in the epigenetic reprogramming and germline development (Andres et al. 2015; Huang et al. 2021; Pembrey et al. 2006; Sandovici et al. 2022; Savva et al. 2021; Shirane and Lorincz 2023; van den Berg and Pinger 2016; Xu et al. 2022). Therefore, alcohol consumption could potentially be exacerbating sex-related differences in the epigenome contributing to the sexual dimorphism particularly seen in the F2. The D_2_ females showed reduced drinking and significantly better performance on the accelerating rotarod while the D_2_ males returned to levels which matched controls. While these provide further evidence for sex-specific epigenetic reprogramming, research tailored to analysing genetic and epigenetic markers is required to understand the processes at play.

### Strengths and limitations

The most important methodological aspects of the study were: (i) A free choice intermittent ethanol access paradigm was used rather than forced ethanol exposure (Finegersh and Homanics 2014; Nieto et al. 2022; Rompala et al. 2017, 2018), which better mimics human patterns of ethanol consumption (Beeler et al. 2019; Hussain et al. 2022; Spoelder et al. 2015) (ii) The males were kept for a full spermatogenesis cycle before mating to minimise direct effects of ethanol on the sperm, thus ensuring that the observed effects were intergenerational. Such an approach has been used by only a few previous studies (Hussain et al. 2022; Nieto et al. 2022). (iii) True transgenerational effects of PPEE on behaviour of offsprings has been shown for the first time in a rat model. (iv) Including both male and female offspring, revealing clear sex differences in some of the inter and transgenerational effects. While a few studies have indicated potential effects (Abel and Lee 1988; Cambiasso et al. 2022) or looked at developmental signs like birthweights and litter effects (Stockard and Papanicolaou 1916a, b) none have looked at behaviours one generation removed from chronic voluntary paternal alcohol intake.

This paper provides a good start for the understanding of transgenerational impacts of PPEE, however, there are a few methodological limitations which must be considered. The IA2BC paradigm required isolation of animals and both rats and humans show social isolation increases ethanol drinking along with anxiety and depression-like behaviours (Kinley et al. 2021; Kokare et al. 2017; Lesscher et al. 2015; Novoa et al. 2021). As such, isolating rats during adolescence might induce epigenetic changes not associated with ethanol intake. We also focussed on the offspring of the highest drinkers which can mask some of the information with regards to moderate drinking levels and assess correlations between quantity of paternal drinking and offspring development.

## Conclusions

This study indicates the vital role paternal ethanol consumption plays in offspring outcomes. The effects seen in this study demonstrate clear epigenetic effects that are sexual dimorphic in nature. Our findings indicate a father’s role in their child’s development is more than just the genes passed down with paternal preconception environment leading to a host of problems in future generations (Bajrami and Spiroski 2016). Future research in this area would lead to a better understanding of the mechanisms underlying this epigenetic inheritance of drug use and how to counter it. At the same time, these results indicate the need to update the general awareness about the effects of alcohol on both parents on future generations.

## Electronic supplementary material

Below is the link to the electronic supplementary material.

Supplementary Material 1

Supplementary Material 2

## Data Availability

The data are available upon request from the authors.

## Abbreviations

D: Drinkers
IA2BC: Intermittent access two bottle choice
ND: Non-Drinkers
PND: Postnatal Day
PPEE: Preconceptual Paternal Ethanol Exposure
