# Supplementary figures and images for: Preconceptual paternal ethanol drinking induces sexually dimorphic behavioural changes across 2 generations
Source: Psychopharmacology (Berl). 2025 May 20;242(11):2447–64. doi: 10.1007/s00213-025-06807-w (PMC12578749; doi:10.1007/s00213-025-06807-w)

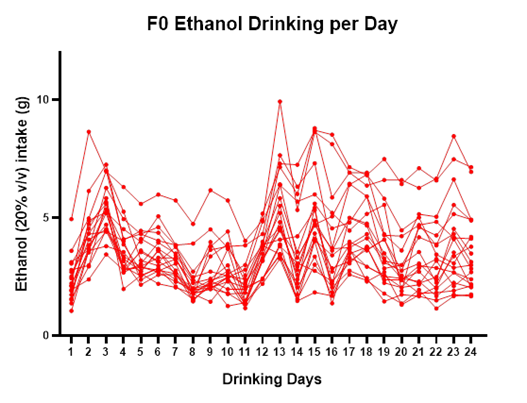

Supplement: Supplementary file 1 — Supplementary Material 1 [file 213_2025_6807_MOESM1_ESM.png]
