# Supplementary material for: Preconceptual paternal ethanol drinking induces sexually dimorphic behavioural changes across 2 generations
Source: Psychopharmacology (Berl). 2025 May 20;242(11):2447–64. doi: 10.1007/s00213-025-06807-w (PMC12578749; doi:10.1007/s00213-025-06807-w)
Supplement: Supplementary file 2 — Supplementary Material 2 [file 213_2025_6807_MOESM2_ESM.docx]

**Supplementary Figures and Tables**

| **Milestone** | **F1** | | | | **F2** | | | |
| --- | --- | --- | --- | --- | --- | --- | --- | --- |
|  | ND_1_ Mean (SD) | D_1_ Mean (SD) | t-value (df) | p-value | ND_2_ Mean (SD) | D_2_ Mean (SD) | t-value (df) | p-value |
| Litter Number | 13.90 (2.73) | 12.40 (4.17) | 0.95 (18) | 0.354 | 13.00 (1.92) | 11.57 (5.03) | 0.70 (12) | 0.496 |
| Birth from Pairing Day | 25.00 (2.67) | 26.60 (4.67) | 0.94 (18) | 0.359 | 26.29 (0.49) | 28.29 (3.95) | 1.33 (12) | 0.208 |
| Deaths PND 0-4 | 0.20 (0.42) | 1.10 (1.85) | 1.50 (18) | 0.152 | 0.00 (0.00) | 1.00 (1.00) | 2.65 (12) | 0.021** |
| Coat Growth | 9.30 (0.95) | 9.44 (0.53) | 0.40 (17) | 0.692 | 9.14 (0.38) | 10.00 (0.71) | 2.74 (10) | 0.021** |
| Eye Opening | 16.10 (0.57) | 16.44 (0.88) | 1.02 (17) | 0.320 | 16.14 (0.38) | 16.80 (0.45) | 2.76 (10) | 0.002** |
| Ear Separation | 13.60 (0.70) | 14.44 (0.73) | 2.58 (17) | 0.019** | 13.71 (0.49) | 14.00 (0.00) | 1.29 (10) | 0.226 |
| Walking | 14.70 (0.48) | 15.11 (0.60) | 1.65 (17) | 0.117 | 14.14 (0.38) | 14.40 (0.55) | 0.97 (10) | 0.356 |
| Rearing | 18.20 (0.42) | 18.44 (0.53) | 1.12 (17) | 0.277 | 18.14 (0.38) | 18.40 (0.55) | 0.97 (10) | 0.356 |
| Self-Grooming | 16.00 (0.47) | 15.67 (1.00) | 0.95 (17) | 0.357 | 14.86 (0.69) | 15.40 (0.55) | 1.46 (10) | 0.176 |

***Table S1. Developmental milestones analysis results***

| **Surface Righting** | | | | | | | | |
| --- | --- | --- | --- | --- | --- | --- | --- | --- |
|  | **Sire** | **Sex** | **Age** | **N** | **Weight (g)** | | **Min time (sec)** | |
| **F1** | Non-Drinker | Female | PND2 | 8 | 8.1 | (1.29) | 10.21 | (5.14) |
|  |  |  | PND4 | 10 | 11.83 | (1.17) | 6.16 | (6.80) |
|  |  | Male | PND2 | 12 | 8.15 | (1.26) | 18.04 | (20.72) |
|  |  |  | PND4 | 10 | 12.83 | (1.43) | 2.84 | (1.71) |
|  | Drinker | Female | PND2 | 8 | 8.29 | (1.03) | 11.95 | (13.49) |
|  |  |  | PND4 | 9 | 11.41 | (0.85) | 3.44 | (2.23) |
|  |  | Male | PND2 | 9 | 9.14 | (1.11) | 4.16 | (1.85) |
|  |  |  | PND4 | 9 | 12.74 | (1.11) | 3.62 | (2.00) |
|  | **Sire** | **Sex** | **Age** | **N** | **Weight (g)** | | **Min time (sec)** | |
| **F2** | Non-Drinker | Female | PND2 | 7 | 9.21 | (1.18) | 15.04 | (18.38) |
|  |  |  | PND4 | 7 | 12.59 | (1.31) | 4.28 | (2.16) |
|  |  | Male | PND2 | 9 | 9.64 | (0.86) | 8.04 | (4.98) |
|  |  |  | PND4 | 7 | 14 | (0.86) | 2.3 | (0.76) |
|  | Drinker | Female | PND2 | 7 | 8.9 | (0.74) | 9.31 | (8.46) |
|  |  |  | PND4 | 7 | 13.7 | (2.77) | 5.2 | (2.13) |
|  |  | Male | PND2 | 11 | 9.77 | (1.70) | 8.97 | (7.73) |
|  |  |  | PND4 | 7 | 15.09 | (2.45) | 3.55 | (2.48) |

*M (SD)*

***Table S2. Means and standard deviations for Surface righting times and weights of the F1 and F2.*** Means and standard deviations for the weights and minimum surface righting times in each group separated by sex, condition and age.

| Locomotor Activity | | | |
| --- | --- | --- | --- |
| F1 | F-statistic | df1, df2 | p-value |
| Distance Travelled | 11.53 | 2, 64 | <.001** |
| Sex | 2.94 | 1, 32 | .096* |
| Sire Condition | 0.08 | 1, 32 | .775 |
| Sex x Sire | 1.18 | 1, 32 | .286 |
| Rearing | 23.96 | 2, 64 | <.001** |
| Sex | 4.92 | 1, 32 | .034** |
| Sire Condition | 3.94 | 1, 32 | .056* |
| Sex x Sire | 0.44 | 1, 32 | .511 |
| F2 | F-statistic | df1, df2 | p-value |
| Distance Travelled | 3.90 | 2, 40 | .028** |
| Sex | 1.13 | 1, 20 | .301 |
| Sire Condition | 0.15 | 1, 20 | .702 |
| Sex x Sire | 0.02 | 1, 20 | .902 |
| Rearing | 13.15 | 2, 40 | <.001** |
| Sex | 0.04 | 1, 20 | .843 |
| Sire Condition | 0.01 | 1, 20 | .913 |
| Sex x Sire | 1.13 | 1, 20 | .301 |

***Table S3. Locomotor activity analysis results***

| Accelerating Rotarod | | | |
| --- | --- | --- | --- |
| F1 | F-statistic | df1, df2 | p-value |
| Latency to Fall |  |  |  |
| Dose | 26.46 | 2, 64 | <.001** |
| Sire | 4.17 | 1,32 | 0.235 |
| Sex | 4.12 | 1, 32 | .051* |
| Dose x Sire | 6.62 | 2, 64 | .002** |
| % Drop in performance |  |  |  |
| Dose | 27.83 | 1, 32 | <.001** |
| Sire | 6.07 | 1, 32 | .019** |
| Sex | 0.08 | 1, 32 | 0.778 |
| F2 | F-statistic | df1, df2 | p-value |
| Latency to Fall |  |  |  |
| Dose | 53.7 | 2, 40 | <.001** |
| Sire | 12.27 | 1, 20 | .002** |
| Sex | 9.3 | 1, 20 | .006** |
| Sire x Sex | 5.38 | 1, 20 | .031** |
| Dose x Sire | 3.14 | 2, 40 | .054* |
| Dose x Sex | 4.48 | 2, 40 | .018** |
| % Drop in performance |  |  |  |
| Dose | 33.44 | 1,20 | <.001** |

***Table S4. Rotarod analysis results***

| Intermittent Ethanol Access | | | |
| --- | --- | --- | --- |
| F1 | F-statistic | df1, df2 | p-value |
| g/kg drinking |  |  |  |
| Drinking days | 8.76 | 23, 119.69 | <.001** |
| Sire | 3.69 | 1, 81.45 | 0.058* |
| Sex | 60.95 | 1, 81.45 | <.001** |
| Sire x Sex | 10.96 | 1, 81.45 | .001** |
| Ethanol Preference |  |  |  |
| Drinking session | 32.24 | 1, 24 | <.001** |
| Drinking session x Sex | 11.50 | 1, 24 | .002** |
| Sex | 6.75 | 1, 24 | .016** |
| Sire x Sex | 6.36 | 1, 24 | .019** |
| F2 | F-statistic | df1, df2 | p-value |
| g/kg drinking |  |  |  |
| Drinking days | 7.86 | 23, 109.30 | <.001** |
| Sire | 8.51 | 1, 49.76 | .005** |
| Sex | 24.81 | 1, 49.76 | <.001** |
| Sire x Sex | 7.25 | 1, 49.76 | .010** |
| Ethanol Preference |  |  |  |
| Sire x Sex | 5.01 | 1, 19 | .037** |

***Table S5. Drinking ethanol and ethanol preference results***

Figure S1. F0 Ethanol consumption across 24 sessions. The top 10 highest cumulative drinkers were taken for mating.
